# Supplementary material for: Subcellular Compartments Interplay for Carbon and Nitrogen Allocation in Chromera velia and Vitrella brassicaformis
Source: Genome Biol Evol. 2019 Jun 13;11(7):1765–79. doi: 10.1093/gbe/evz123 (PMC6668581; doi:10.1093/gbe/evz123)
Supplement: Supplementary_Material_evz123 [file supplementary_material_evz123.zip › SUPPLEMENTARY MATERIAL legends.docx]

SUPPLEMENTARY MATERIAL:

**Supplementary Table S1: Reference protein lists grouped by metabolic function; inferred phylogenies of argJ, polyprenyl-PP synthases, and PRPP synthase; set of updated reference protein sequences.** Trees in Newick format, accessions are marked on leaves. The maximum-likelihood trees were inferred by the IQ-TREE software (see main text Methods). Sequence updates are based on homology searches within alternative transcriptomic datasets (elongated sequences) and comparison of orthologous sequences in *C. velia* and *V. brassicaformis* (merged contigs).

**Supplementary Table S2: Prediction scores for reference proteins and the species-specific ASAFind scoring matrices of *C. velia* and *V. brassicaformis*.**

**Supplementary Figure S3: Targeting peptide score distributions for reference sequences.** Reference colors: green – plastid, blue – mitochondrial, grey – other (cytosolic or non-plastid endomembrane route).
