# Supplementary figures and images for: Subcellular Compartments Interplay for Carbon and Nitrogen Allocation in Chromera velia and Vitrella brassicaformis
Source: Genome Biol Evol. 2019 Jun 13;11(7):1765–79. doi: 10.1093/gbe/evz123 (PMC6668581; doi:10.1093/gbe/evz123)

Plastid predictors:


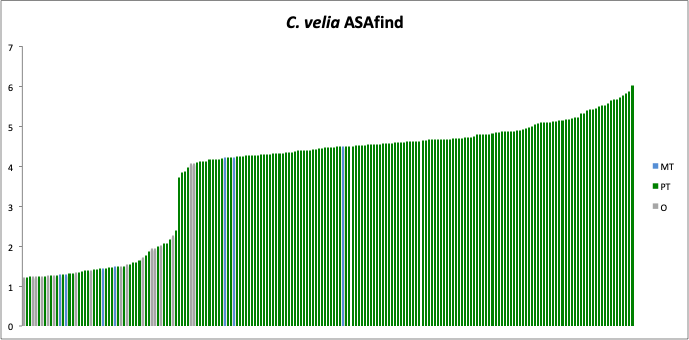

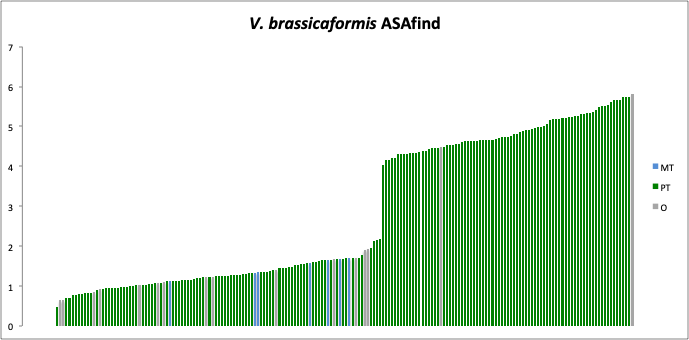


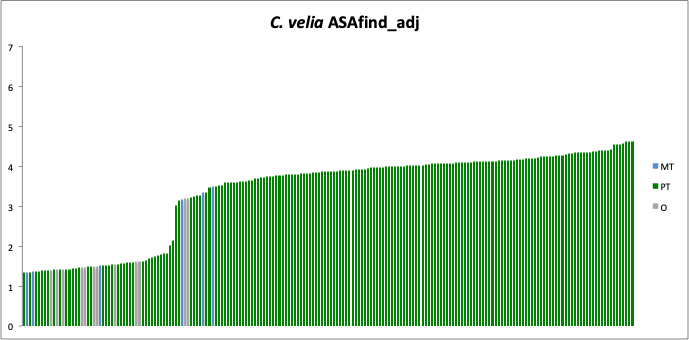

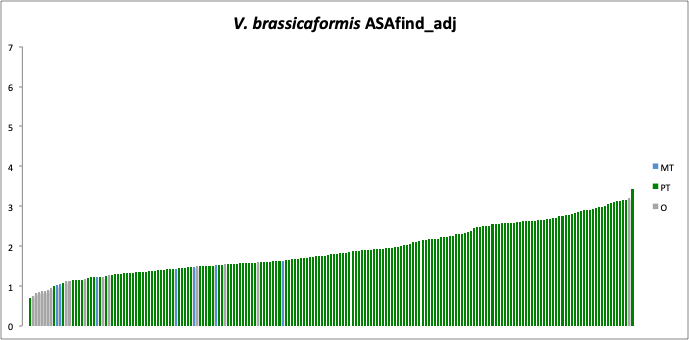


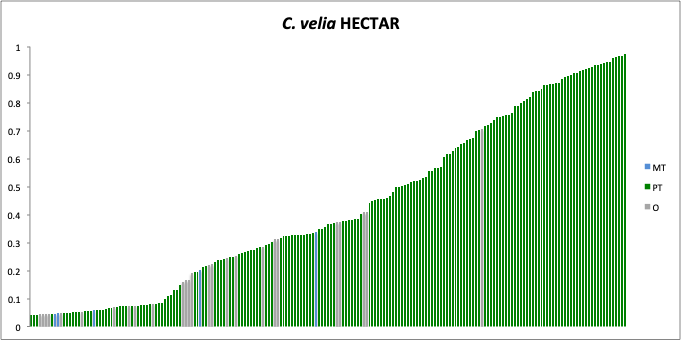

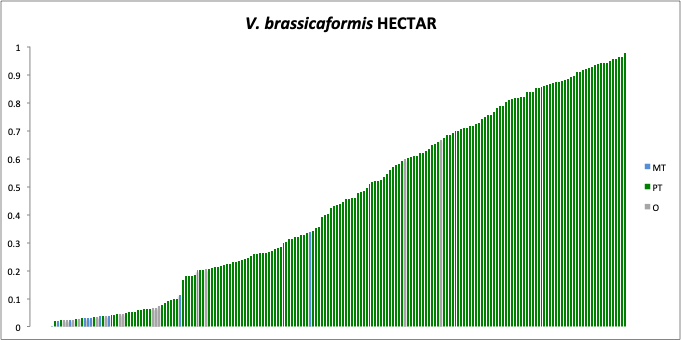


Mitochondrial predictors:


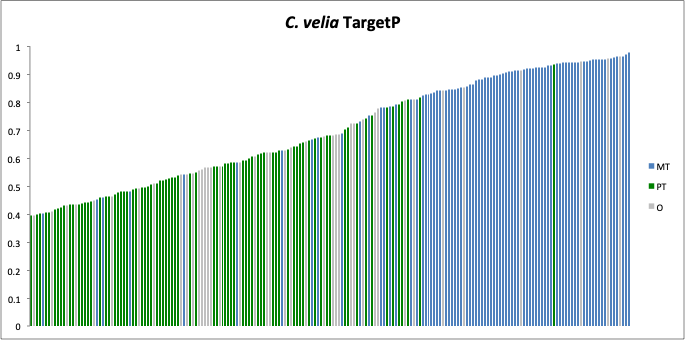

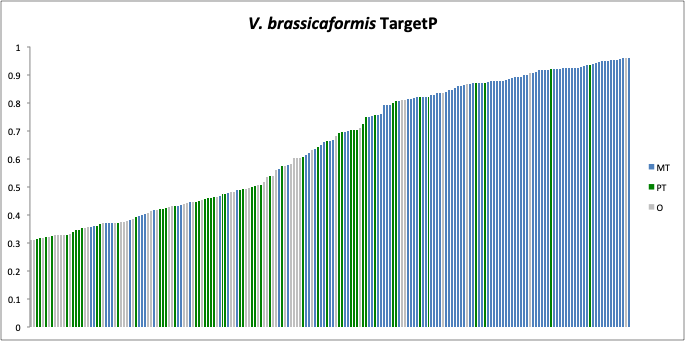


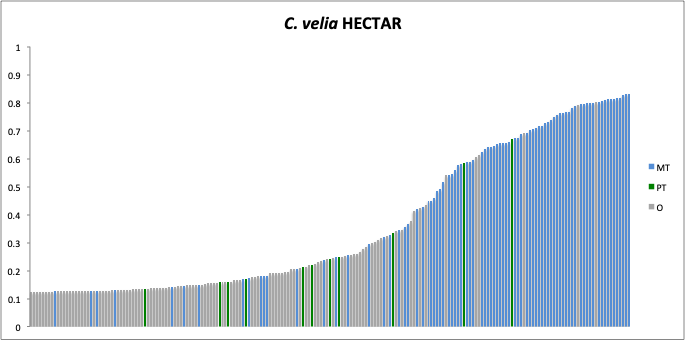

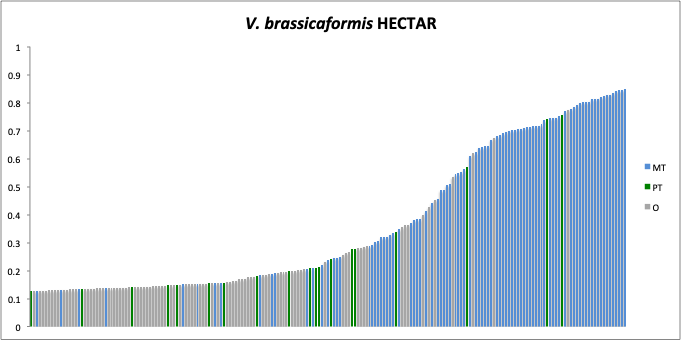


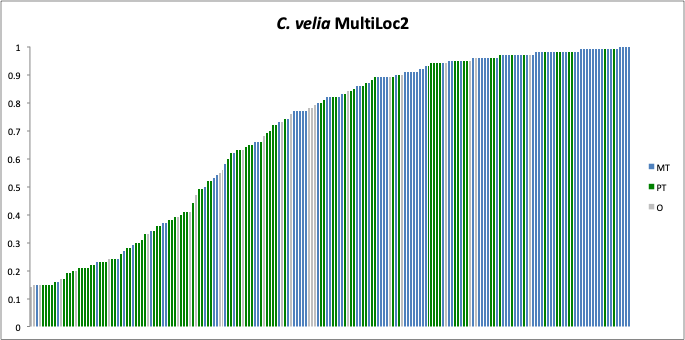

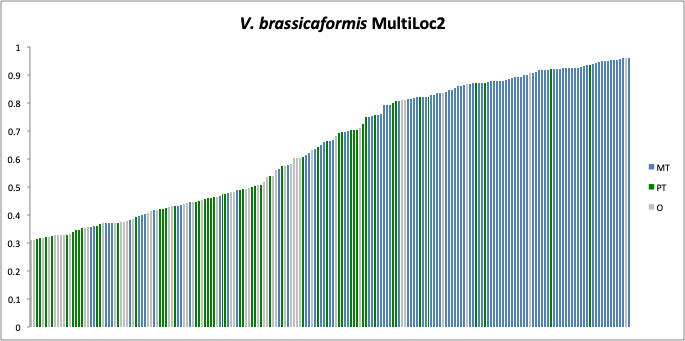

Supplement: Supplementary_Material_evz123 [file supplementary_material_evz123.zip › Supplementary Figure S3.docx]
